# A COVID-19 first evaluation clinic at a university hospital in Turkey

**DOI:** 10.3906/sag-2104-152

**Published:** 2021-09-07

**Authors:** Gülçin TELLİ DİZMAN, Gökhan METAN, Çağlayan Merve AYAZ CEYLAN, Hayriye ALTUNAY, Mertcan UZUN, Gamze GÜRSOY, Zahit TAŞ, Gizem KARAHAN, Farida AHMADOVA, Tuğba SARICAOĞLU, Zeynep Cansu ÇALIŞKAN, Alpaslan ALP, Meliha Çağla SÖNMEZER, Ahmet Çağkan İNKAYA, ER Ahmet Görkem, Şehnaz ÖZYAVUZ ALP, Oğuz Abdullah UYAROĞLU, Mine DURUSU TANRIÖVER, Nursel ÇALIK BAŞARAN, Gamze DURHAN, Figen DEMİRKAZIK, Ömrüm UZUN, Murat AKOVA, Serhat ÜNAL

**Affiliations:** 1 Department of Infectious Diseases and Clinical Microbiology, Faculty of Medicine, Hacettepe University, Ankara, Turkey; 2 Infection Control Committee, Hospitals of Faculty of Medicine, Hacettepe University, Ankara, Turkey; 3 Department of Infectious Diseases and Clinical Microbiology, Ankara City Hospital, Ankara, Turkey; 4 Department of Infectious Diseases, Siirt Training and Research Hospital, Siirt, Turkey; 5 Department of Medical Microbiology, Faculty of Medicine, Hacettepe University Ankara, Turkey; 6 Department of Internal Medicine, Faculty of Medicine, Hacettepe University, Ankara, Turkey; 7 Department of Radiology, Faculty of Medicine, Hacettepe University, Ankara, Turkey

**Keywords:** Severe acute respiratory syndrome coronavirus 2, coronavirus disease 2019, SARS-CoV-2, COVID-19, outpatient clinic, Turkey

## Abstract

**Background/aim:**

We aimed to analyze the usefulness of such a reserved area for the admission of the patients’ symptoms suggesting COVID-19 and compare the demographic and clinical characteristics of the patients with COVID-19 and without COVID-19 who were admitted to C1 during the first month of the COVID-19 outbreak in our hospital.

**Materials and methods:**

A new area was set up in Hacettepe University Adult Hospital to limit the contact of COVID-19 suspicious patients with other patients, which was named as COVID-19 First Evaluation Outpatient Clinic (C1). C1 had eight isolation rooms and two sampling rooms for SARS-CoV-2 polymerase-chain-reaction (PCR). All rooms were negative-pressurized. Patients who had symptoms that were compatible with COVID-19 were referred to C1 from pretriage areas. All staff received training for the appropriate use of personal protective equipment and were visited daily by the Infection Prevention and Control team.

**Results:**

One hundred and ninety-eight (29.4%) of 673 patients who were admitted to C1were diagnosed with COVID-19 between March 20, 2020, and April 19, 2020. SARS-CoV-2 PCR was positive in 142 out of 673 patients. Chest computerized tomography (CT) was performed in 421 patients and COVID-19 was diagnosed in 56 of them based on CT findings despite negative PCR. Four hundred and ninety-three patients were tested for other viral and bacterial infections with multiplex real-time reverse-transcriptase PCR (RT-PCR). Blood tests that included complete blood count, renal and liver functions, d-dimer levels, ferritin, C- reactive protein, and procalcitonin were performed in 593 patients. Only one out of 44 healthcare workers who worked at C1 was infected by SARS-CoV-2.

**Conclusion:**

A well-planned outpatient care area and teamwork including internal medicine, microbiology, and radiology specialists under the supervision of infectious diseases specialists allowed adequate management of the mild-to-moderate patients with suspicion of COVID-19.

## 1. Introduction

The rapid dissemination of novel severe acute respiratory syndrome coronavirus 2 (SARS-CoV-2) became a big challenge for the appropriate management of coronavirus disease 2019 (COVID-19) [1, 2].The total number of patients with COVID-19 reached up to 80,794,084 with 1,766,513 deaths on 27 December 2020^1^.The clinical spectrum of COVID-19 varies from asymptomatic infection to respiratory failure, septic shock, and multiple organ dysfunction syndromes (MODS) and death [3, 4]. In a study from Wuhan; 81% of patients had mild disease, 14 % of patients had severe disease, and 5 % had the critical disease [5].

A large number of the patients who were admitted to hospitals with suspicion of COVID-19 overcrowded the emergency rooms (ER), which can lead to delay in the assessment of other emergencies and, also, pose a high risk for transmission of COVID-19 during triage in the ER [6]. Setting up a rapid response infrastructure was able to reduce the workload in the ER [7, 8].

There were 148,817 cases with 4636 deaths around the world when the first case of COVID-19 was detected on 11 March 2020 in Turkey and on 21 March 2020 in Hacettepe University Hospitals^1^ [9].The spread of SARS-CoV-2 in ER is of concern and a new area was set up in our hospital to limit the contact of COVID-19 suspicious patients with other patients, which was named COVID-19 First Evaluation Outpatient Clinic (C1). Here, we aimed to analyze the usefulness of such a reserved area for the admission of the patients’ symptoms suggesting COVID-19 and compare the demographic and clinical characteristics of the patients with COVID-19 and without COVID-19 who were admitted to C1 during the first month of the COVID-19 outbreak in our hospital.

## 2. Materials and methods

Hacettepe University Adult Hospital is a 730-bed tertiary care center in Ankara, the capital city of Turkey. C1 was established at Hacettepe University Adult Hospital immediately after the detection of the first cases in Turkey. Any patient who was admitted to the hospital was questioned for COVID-19 symptoms and history of contact with a COVID-19 patient at the pretriage areas located at the entrances of the hospital. In case of suspicion for COVID-19, patients were referred to C1. This area was previously used as ER and replaced by the new ER two and a half years ago. C1 consisted of eight single-patient rooms and two sampling rooms, which were all negative-pressurized (Figure). Rooms were periodically checked for air pressure by construction and technical services.

Patients who were suspected of COVID-19 according to the World Health Organization (WHO) and the Turkish Ministry of Health COVID-19 guidelines were taken into the sampling room^2^. Vital signs were checked before sampling. Hypoxic (pO_2_ < 93 at ambient air) and tachypneic (respiratory rate > 22/min) patients were referred to the ER. A combined oropharyngeal and nasopharyngeal sample was taken by a doctor for SARS-CoV-2 real-time reverse-transcriptase–polymerase-chain-reaction (RT-PCR) and a blood sample was taken by the nurse in another room if requested by the attending physician according to the clinical presentation and local guideline. Viral nucleic acid isolation from the samples was achieved by using Bio-Speedy vNAT viral nucleic acid buffer (Bioeksen R&D Technologies Ltd, Turkey). SARS-CoV-2 RT-PCR kit (Bioeksen R&D Technologies Ltd, Turkey) was used for the diagnosis in our hospital. The results were available on the same day. Multiplex RT-PCR was performed by using the Allplex Respiratory Panel (Seegene, South Korea) to detect other respiratory viral and bacterial infections.

Chest computerized tomography (CT) was performed at the area reserved for COVID-19 patients in the radiology unit which was localized just behind the C1. Patients were followed up in the solitary rooms until results were obtained. Appropriate personal protective equipment for aerosol-generating procedures was used either during sampling or examining the patients according to the instructions. Sampling rooms were cleaned and then disinfected by hydrogen peroxide between each patient after every procedure. Patients with suspicion of COVID-19 were hospitalized in the wards reserved for COVID-19 or discharged for home isolation according to the local guidelines. The hospitalization criteria were as follows; age higher than 50 years, presence of comorbid diseases such as diabetes mellitus, chronic pulmonary diseases, hypertension, cancer, stem cell or solid organ transplantation, detection of pneumonia, increase in lactate dehydrogenase (LDH), C-reactive protein (CRP), neutrophil-to-lymphocyte ratio (NLR), D-dimer, ferritin, cardiac enzymes, and acute renal failure (Supplementary Table 1). Electrocardiography (ECG) was performed if antiviral therapy with hydroxychloroquine (HQ) was prescribed for those who were followed up at home isolation (Supplementary Table 2).

A total of 20 physicians, 12 nurses, and 12 housekeeping staff worked at C1.The staff worked in three shifts which consisted of 8 or 16 h. Four physicians, 4 (2 at night shifts) nurses, and 3 (2 at night shifts) housekeeping staff worked at each shift. The resting period was 16 or 24 h. A team of physicians from various medical departments took part in the evaluation of patients. All of the C1 staff received training about the appropriate use of personal protective equipment and as well as about the local guideline for the initial management of COVID-19. All procedures were performed under the leadership and supervision of infectious diseases specialists. The outpatient clinic was available 24 h a day including the weekends. All healthcare workers were monitored daily for any symptom suggesting COVID-19, and SARS-CoV-2 RT-PCR was performed in case of suspicion of COVID-19. However, there was no routine laboratory-based screening policy for healthcare workers including C1 staff to detect possible asymptomatic cases except close contacts of the patients with COVID-19.

The characteristics of the patients who were admitted to C1 between March 20, 2020, and April 19, 2020, were evaluated, retrospectively. Demographic data such as age, sex, occupation as being a healthcare worker, comorbidities, clinical signs, or symptoms and contact history with a confirmed COVID-19 case were recorded. The results of complete blood count (CBC), coagulation parameters, liver and renal functions, CRP, procalcitonin (PCT), LDH, and creatine kinase levels which were performed according to the local guidelines were extracted from the hospital database.

The case identification was based on PCR results and radiological findings. Patients with a positive SARS-CoV-2 PCR were identified as proven COVID-19, patients with clinical symptoms with a positive chest CT were identified as probable COVID-19, and patients who had an alternative diagnosis with a negative SARS-CoV-2 PCR and/or chest CT were grouped as non-COVID-19 patients.

Patients with COVID-19 (proven and probable patients) were compared with non-COVID-19 patients in terms of demographic and clinical characteristics. Categorical variables were presented as numbers and percentages. Continuous variables were given as mean and standard deviation (SD) if normally distributed, median and minumm–maximum (min–max) if nonnormally distributed. Categorical variables were compared using the Pearson chi-square test/Fisher’s exact test. Continuous variables were compared using the Student’s *t*-test or Mann–Whitney *U* test according to the distribution of variables. p-values less than 0.05 were considered statistically significant. For all statistical analyses, IBM SPSS Statistics 24 v17.0 (IBM Corp. Chicago, IL, USA) software was used.

The study was approved by the Institutional Non-Interventional Clinical Research Ethics Committee, Ankara, Turkey.

## 3. Results

The median age of the patients was 35 years. Patients with COVID-19 were older than non-COVID-19 patients (38 vs 35 years, p = 0.002). Three hundred fifty-six (52.9 %) patients were female and 308 (45.8 %) patients were healthcare workers (Table 1). A total of 198 (29.4%) of 673 patients who were admitted to C1 were diagnosed with COVID-19. SARS-CoV-2 PCR was positive in 142 (71.7%) of 198 patients.

Hypertension and diabetes mellitus were the most common comorbid diseases. Three hundred eighty-one (56.6%) patients had a history of contact with a COVID-19 patient. Contact history was more common in patients with COVID-19 (67.7% vs 52%, p = 0.001). Cough (73.2% vs 65.5, p = 0.05), myalgia/arthralgia (42.4% vs 22.7%, p = 0.001), and loss of smell and/or taste (6.6% vs 2.1%) were more common in COVID-19 patients, and sore throat was more common in non-COVID-19 patients (26.9% vs 19.7, p = 0.047). The rates of fever, dyspnea, diarrhea, and runny nose during admission were similar in the two groups (Table 2).

Lymphocytopenia, thrombocytopenia, and high ferritin levels were more common in patients with COVID-19. CRP levels were higher in patients with COVID-19 than non-COVID-19 patients (p = 0.001), and PCT levels were lower in patients with COVID-19 than non-COVID-19 patients (p = 0.011) (Table 3).

A chest X-ray was performed in 518 patients, and chest CT was performed in 421 (62.6%) patients. Pneumonia was detected at the chest CT of 139 patients and the chest X-ray of 5 patients. SARS-CoV-2 PCR was positive in 83 (59.71%) of 139 patients who had a chest CT that was reported as compatible with COVID-19 pneumonia. All of the patients who had an X-ray compatible with COVID-19 pneumonia had also positive SARS-CoV-2 PCR.

Multiplex PCR for other viral pathogens were found to be positive in 27 (7.7%) of non-COVID-19 patients while only two (1.3%) of 152 patients with COVID-19 (p = 0.005). The rate of positive multiplex PCR for bacterial pathogens was similar between patients with COVID-19 and non-COVID-19 patients (Table 3). The most common bacteria that was detected in multiplex PCR was *Haemophilus influenza* and, the most common virus was rhinovirus (Table 4).

Two hundred and six (30.61 %) of the patients who were admitted to C1 were hospitalized at COVID-19 wards; 162 (24.07 %) of these were diagnosed with COVID-19. In 44 (6,54%) of 206 patients, COVID-19 was ruled out after hospitalization. Thirty-six (5.35 %) patients were followed up as outpatient [10]. Hydroxychloroquine (HQ) was given as monotherapy to patients with symptoms without pneumonia. Patients with pneumonia were treated with a combination of HQ plus azithromycin as inpatients. Nonsteroidal antiinflammatory drugs or paracetamol were prescribed for fever. Antibiotics were administered for 16 patients with COVID-19 in whom pneumonia was detected at chest CT. CRP levels were significantly higher in these patients (p = 0.001). The 22 out of 673 patients who received an antibacterial treatment had higher CRP and PCT levels when compared with the patients who did not receive antibacterial treatment (Table 5).

A total of 673 SARS-CoV-2 RT-PCR tests were performed in our hospital from March 20, 2020 to April 19, 2020.Three hundred and eight of these tests were performed for healthcare personnel who had symptoms compatible with COVID-19 or exposed to COVID-19 patients without appropriate personal protective equipment.

Eighty-two (41.4 %) of 198 patients that were diagnosed with COVID-19 were healthcare personnel. COVID-19 was diagnosed in only one out of 44 staff who worked at C1.This was an internist who reported to have close contact with two internists who were the first COVID-19 patients at our hospital.

## 4. Discussion

Immediately after detection of COVID-19 in Turkey, the first evaluation outpatient clinic was set up to avoid the chaos which can occur with the increase in the number of cases. The separate area for the evaluation of the patients with suspicion of COVID-19 became an important advantage at our hospital in terms of preventing the spread of SARS-CoV-2 infection, detailed medical investigation of all patients who were admitted to C1 and timely diagnosis and management of COVID-19 patients.

The 45.8% of 673 patients who were admitted to C1 were healthcare workers. The risk of COVID-19 is high in healthcare workers due to repeated exposures with COVID-19 patients; however, the rate of COVID-19 was not higher in healthcare workers than in the other people who were admitted to C1 during the first month of the pandemic at our hospital. The concerns about the disease and encouraging policy to seek medical investigation for COVID-19 as early as possible can have a role in the high number of admissions by healthcare workers. According to the local hospital instructions, any healthcare worker who had symptoms that can be related to COVID-19 was strongly recommended to admit to C1 for evaluation.

Advanced age is considered an important risk factor for COVID-19 [7, 11]. In a study from Wuhan, the median age of the patients with COVID-19 was reported as 60 years, ranging from 18 to 95 years, and 38.3% of the patients were older than 65 years [11]. Similar findings were reported by a retrospective cohort study from France which included 89,530 patients [12]. Although the patients with COVID-19 who were diagnosed at C1 during the first month of the pandemic were younger when compared with other countries [13, 14], the patients with COVID-19 were older than patients without COVID-19 (38 years vs 35 years, p = 0.002). The curfew for people who were older than 65 years was probably the main reason which limited the spread of COVID-19 in elderly patients during the first month of the pandemic in Turkey.

The presence of comorbid diseases can influence the outcome of COVID-19 patients. In a metaanalysis, hypertension was the most prevalent comorbid disease with a rate of 21.1% of 1576 patients. Diabetes mellitus, cardiovascular disease, and respiratory system disease were the other common comorbidities in 9.7%, 8.4%, and 1.5% of the patients, respectively [15]. Thirty-one point five percent of the patients who were admitted to C1 had comorbid diseases, and the most common diseases were diabetes mellitus and hypertension, as observed in the previous studies. Although chronic diseases were detected more in patients with COVID-19, no statistically significant difference was detected between the two groups.

Fever, cough, diarrhea, headache, and dyspnea were the most common clinical symptoms in a metaanalysis that included 45 studies [16]. The rates of cough, myalgia/arthralgia, sore throat, and loss of smell or/and taste were more prevalent in patients with COVID-19, and the presence of these symptoms should trigger SARS-CoV-2 PCR. Our local guideline recommended chest CT for patients with comorbid diseases and/or respiratory symptoms (cough, fever, etc.) in suspicion of COVID-19. A total of 421 chest CTs were performed and this approach allowed to diagnose 56 patients whose SARS-CoV-2 PCR tests were negative but CT findings were concordant with COVID-19. In a recent study, bronchoalveolar lavage fluid was found as positive for SARS-CoV-2 PCR in 18 patients who had two negative consecutive PCR tests performed from a nasopharyngeal swab. Chest CT scans were compatible with COVID-19 in those patients [17].

High D-dimer levels and lymphopenia were reported to be related to poor outcomes [18–20]. We were able to test CBC in 571 patients and D-dimer in 353 patients. Lymphocyte and platelet counts were found to be statistically significantly lower in those diagnosed with COVID-19 compared to those who were not. D-dimer values did not differ between the two groups but ferritin values were found to be significantly higher in patients with COVID-19.

Fever is a common symptom of respiratory tract infections. Forty-seven percent of 673 patients who were admitted to C1 had a fever. It is important to rule out bacterial infections in patients presenting with respiratory symptoms and fever. We were able to request multiplex PCR for bacterial respiratory pathogens in 493 patients. Ninety-six of 493 PCR tests were reported as positive. However, the rate of positivity of the multiplex PCR panel for respiratory bacterial pathogens was not significantly different in patients with COVID-19 when compared with non-COVID-19 patients (%17.3 vs %20.4, p = 0.428). The most commonly detected bacterial pathogen *was H. influenza* in 12 patients with COVID-19. A recent metaanalysis that included 24 studies reported that 3.5% of the patients with COVID-19 had a bacterial coinfection. The rate was highly variable as 0% to 42%. However, 71.9% of patients with COVID-19 received antibiotics in the same analysis [21], while 10.6% out of 198 patients with COVID-19 received an antibacterial agent in our cohort. A total of 130 out of 198 patients with COVID-19 received azithromycin in our cohort. One can speculate that azithromycin use should be regarded as antibacterial therapy for possible community-acquired bacterial pneumonia. However, in our hospital it was used in combination with hydroxychloroquine as a part of COVID-19 treatment according to Turkish Ministry of Health treatment guidelines^2^. In a study from Italy, overall antibiotic consumption in March–April 2020 was not different from that in the prepandemic period but it was found that the mean consumption of levofloxacin and ceftriaxone was as high as azithromycin which was not the case in our hospital [22]. Nevertheless, use of unnecessary antibiotics including azithromycin can influence the antibacterial resistance and should be avoided [23]. The reason for the low rate of prescribing antibiotics targeting community-acquired pneumonia such as respiratory quinolones or ceftriaxone can be rapid access to SARS-CoV-2 PCR and chest CT on the same day of admission. Multiplex PCR for bacterial pathogens was reported usually in 2 days. Patients who had a positive bacteria PCR result but did not receive empirical antibiotics were reevaluated by infectious diseases specialists. In case of resolution of the symptoms, antibacterial therapy was not recommended. Evaluation of clinical, radiological, and microbiological results together resulted in a more adequate antibiotic usage.

The biomarkers such as CRP and PCT can also guide to initiate antibiotics while waiting for PCR results and they have been widely investigated in the differential diagnosis of community-acquired pneumonia and sepsis, previously [24, 25]. The mean CRP and PCT values of the patients who received antibacterial agents were found to be significantly higher than the patients who did not receive antibiotics (Table 5). However, mean CRP levels were higher in patients with COVID-19. High CRP levels were reported in patients with COVID-19 and reported to be related to poor prognosis [18–20]. In the patients who were admitted to C1, mean CRP levels were significantly higher in COVID-19 patients with pneumonia when compared with COVID-19 patients without pneumonia (3.0543 ± 4.379 vs 0.9103 ± 1.662, p = 0.001). This may reflect disease severity as previously reported [26]. PCT was measured in 296 patients at admission and PCT values were found to be significantly higher in patients without COVID-19.

In a recent guideline about using antibacterial therapy in patients with COVID-19; routine antibacterial treatment is not recommended. Antibacterial treatment is recommended if radiological findings and inflammatory markers are compatible with bacterial coinfection [27]. However, the findings of our study showed that interpretation of the biomarker levels is not quite easy and the role of CRP and PCT can be limited for the differential diagnosis of COVID-19 with other respiratory infections. Furthermore, well-planned studies are required to understand the role of biomarkers to guide antibacterial treatment in this setting.

Other viral infections can mimic COVID-19. Multiplex PCR for viral respiratory pathogens can be useful. Twenty-nine of 503 PCR tests for viral pathogens were positive. The rate of positive PCR for viral pathogens was significantly higher in patients without COVID-19. Coinfection with COVID-19 was observed in only two patients. Human rhinovirus was the most common agent detected in the viral panel. Since the influenza season was still going on, influenza B was the other common pathogen. Adenovirus, RSV, and parainfluenza were the other viruses detected in the viral panel. Coinfection with another virus has been rarely reported in patients with COVID-19 and rhinovirus was reported as the most frequent virus in patients with a negative SARS-CoV-2 PCR [28]. During the early days of the pandemic in Turkey, coinfection with influenza was of concern. A high rate of patients who were admitted to C1 received oseltamivir and the rate of oseltamivir treatment was higher in patients with COVID-19 (93 (47 %) vs 24 (5.05 %), p = 0.001).

In a study from a tertiary care university hospital from Turkey that investigated the risk factors for transmission of COVID-19 among healthcare workers; infection rate of healthcare workers who worked in COVID-19 units was significantly higher than those who did not (8.3% vs 3.4% p *=* 0.027) [31]. Only one out of 44 healthcare workers who worked at C1 was diagnosed with COVID-19. Similarly, in a hospital in South Korea that had a reserved area for COVID-19 patients like C1, transmission of SARS-CoV-2 between healthcare workers and patients was not detected [8]. Hand hygiene, using of appropriate personal protective equipment, monitoring of patients in isolated single rooms and, implementation of cleaning and disinfection in all rooms after each patient protected healthcare workers as well as patients from COVID-19 transmission. Beside the well-designed physical infrastructure, it is important to keep the working hours of the staff optimal to maintain the compliance with safe working instructions in a high-risk area.

## 5. Conclusions

Early diagnosis of infected patients and ensuring adequate isolation are very important to control the spread of COVID-19. The purpose of setting up the COVID-19 first evaluation outpatient clinic was to prevent the overcrowding of ER due to mild or moderate infections, ensure appropriate distancing and isolation, and enable emergency services to serve for real emergencies. A well-planned outpatient care area and teamwork including internal medicine, microbiology, and radiology specialists under the supervision of infectious diseases specialists allowed adequate management of the mild-to-moderate patients with suspicion of COVID-19.

## Supplementary Information





## Figures and Tables

**Figure:**
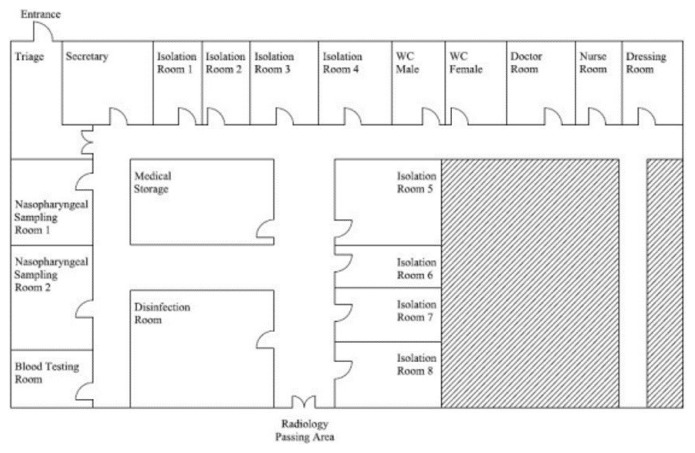
COVID-19 First Evaluation Clinic.

**Table 1:** Comparison of demographic characteristics of the patients who were admitted to “C1 First Evaluation Clinic”.

Characteristic	All patients (N: 673)	Proven and probable COVID-19 (N: 198)	Without COVID-19 (N: 475)	p-value
Age (years) Median (min–max)	35 (18–90)	38 (19–77)	35 (18–90)	*0.002* **
Gender n (%) Male	317 (47.1)	94(47.5)	223 (46.9)	0.901***
Sex n (%) Female	356 (52.9)	104 (52.5)	252 (53.1)	0.901***
Healthcare worker n (%)	308 (45.8)	82 (41.4)	226 (47.6)	0.144***
Current smoking n (%)	145 (21.5)	44 (28.9)	101 (35.7)	0.167***
History of contact with a diagnosed COVID-19 case n (%)	381(56.6)	134 (67.7)	247 (52)	*0.001* ***
Chronic obstructive pulmonary disease n (%)	212 (31.5)	68 (34.3)	144 (30.3)	0.305***
Hypertension n (%)	45 (6.7)	10 (5.1)	35 (7.4)	0.273***
Diabetes mellitus n (%)	31 (4.6)	8 (4)	23 (4.8)	0.651***
Solid malignancy n (%)	17 (2.5)	6 (3)	11 (2.3)	0.590***
Hematological malignancy n (%)	3 (0.4)	0 (0)	3 (0.6)	0.262***
Chronic heart disease n (%)	27 (4)	10 (5.1)	17 (3.6)	0.375***

Italics represent statistically significant results. Median (min–max) was given.

** Mann–Whitney U test

*** Pearson’s chi-square/Fisher’s exact test were used.

**Table 2:** Comparison of symptoms of the patients who were diagnosed with COVID-19 with those of the patients in whom COVID-19 was ruled out.

Symptoms	All patients (N: 673)	Proven and probable COVID-19 (N:198)	Without COVID-19 (N:475)	p-value
Fever (body temperature ≥ 37.5°C) (%)	316 (47)	102 (51.5)	214 (45.1)	0.126***
Cough (%)	456 (67.8)	145 (73.2)	311 (65.5)	*0.050* ***
Myalgia/arthralgia (%)	192(28.5)	84 (42.4)	108 (22.7)	*0.001* ***
Sore throat (%)	167 (24.8)	39 (19.7)	128 (26.9)	*0.047* ***
Loss of smell and/or taste (%)	23 (3.4)	13 (6.6)	10 (2.1)	*0.004* ***
Runny nose (%)	23 (3.4)	8 (4)	15 (3.2)	0.566***
Dyspnea (%)	178 (26.4)	51 (25.8)	127 (26.7)	0.793***
Diarrhea (%)	46 (6.8)	12 (6.1)	34 (7.2)	0.607***

Italics represent statistically significant results.

*** Pearson’s chi-square/Fisher’s exact test was used.

**Table 3:** Comparison of laboratory findings of the patients who were diagnosed with COVID-19 with those of patients in whom COVID-19 was ruled out.

Laboratory findings	All patients	Proven and probable COVID-19	Without COVID-19	p-value
Lymphocyte count mm^3^ (mean ± SD)(N: 571)	1866 ± 1035 (571)	1401 ± 608 (191)	2100 ± 1124 (380)	*0.001* *
Thrombocyte count mm^3^ (mean ± SD) (N: 571)	221,207 ± 60,459 (571)	196,073 ± 49,773 (191)	233,839 ± 61,458 (380)	*0.001* *
C-reavtive protein mg/dl Median (min–max) (N: 423)	0.65(0.03–34.6) (423)	0.94(0.1–24.2) (159)	0.47(0.03–34.6) (264)	*0.001* **
Procalcitonin ng/ml Median (min–max) (N: 296)	0.02 (0–1.95) (296)	0.03 (0.01–0.67) (158)	0.02 (0–1.95) (138)	*0.011* **
Ferritin μg Median (min-max) N: 353)	51 (2.4–1901) (353)	57.7 (2.4–1901) (165)	43.85(2.6–1036.2) (188)	*0.016* **
Lactate dehydrogenase IU/l Median (min–max) (N: 299)	182 (79–4041) (299)	188 (79–4041) (156)	180 (103–1106) (143)	0.135**
Creatine kinase IU/l Median (min–max) (N: 272)	91(2–3249) (272)	91(2–3249) (147)	88 (15–1031) (125)	0.513**
D-dimer (mg/L) (mean ± SD) (N: 383)	0.6 ± 1.32 (383)	0.67 ± 1.79 (171)	0.54 ± 0.75 (212)	0.331*
Alanine aminotransferase IU/L Median (min–max) (N: 325)	20(7–244) (325)	20(8–244) (167)	20.5 (7–210) (158)	0.749**
Creatinine mg/dL Median (min–max) (N: 394)	0.71(0.35–7.41) (394)	0.7(0.37–1.96) (176)	0.72(0.35–7.41) (218)	0.335**
Positive respiratory virus panel (%) (N: 504)	29 (5.75) (504)	2 (1.3) (152)	27 (7.7) (352)	*0.005* ***
Positive respiratory bacterial panel (%) (N: 493)	96 (19.47) (493)	26 (17.3) (150)	70 (20.4) (343)	0.428***

Italics represent statistically significant results. Mean ± SD and median (min–max) were given.

* Student’s *t*-test,

** Mann–Whitney *U* test,

*** Pearson’s chi-square/Fisher’s exact test were used.

**Table 4:** Distribution of multiplex PCR results, antimicrobial treatment choices, C-reactive protein, and procalcitonin levels of the patients who were admitted to “C1 First Evaluation Clinic”.

	COVID-19 with pneumonia (n: 144)	COVID-19 without pneumonia (n: 54)	Non-COVID-19 (n: 475)
**Bacterial multiplex PCR**	**n: 123**	**n: 28**	**n: 342**
*Haemophilus influenzae*	10 (8.13 %)	4 (14.3 %)	47 (13.74 %)
*Streptococcus pneumoniae*	6 (4.88 %)	2 (7.14 %)	14 (4.1 %)
*Haemophilus influenza* and *Streptococcus pneumoniae*	2 (1.63 %)	-	7 (2.05 %)
*Mycoplasma pneumoniae*	1 (0.81 %)	-	-
*Chlamydia pneumoniae*	1 (0.81 %)	-	1 (0.3 %)
*Chlamydia pneumoniae* and *Streptococcus pneumoniae*	-	-	1 (0.3 %)
Total	20 (16.27 %)	6 (21.43 % )	70 (20.47 %)
**Viral multiplex PCR**	**Performed in 124 patients**	**Performed in 28 patients**	**Performed in 353 patients**
Human rhinovirus	-	-	13 (3.68 %)
Adenovirus	1 (4.2 %)	-	6 (1.7 %)
Influenza B	-	1 (3.57 %)	5 (1.42 %)
Parainfluenza 2	-	-	1 (0.28 %)
RSV*-A	-	-	1 (0.28 %)
RSV*-B	-	-	1 (0.28 %)
**Total**	1 (4.2 %)	1 (3.57 %)	27 (7.65 %)
**Antiviral treatment**			
Hydroxychloroquine sulfate	5 (3.5 %)	41 (75.9 %)	
Hydroxychloroquine sulfate plus azithromycin	127 (88.2 %)	3 (5.56 %)	-
Oseltamivir	83 (57.64 %)	10 (18.52 %)	24 (5.05 %)

* Respiratory syncytial virus

**Table 5:** Comparison of procalcitonin and C-reactive protein levels according to receipt of antibacterial treatment.

	Patients who received antibiotics (n = 22)	Patients who did not receive antibiotics (n = 651)	p-value
Procalcitonin (ng/mL) Median (min–max)	0.45 (0.01–0.67)	0.02 (0–1.95)	*0.002* **
C Reactive Protein (mg/dL) Median (min–max)	3.25 (0.21–24.2)	0.61 (0.03–34.6)	*0.001* **

Italics represent statistically significant results. Median (min–max) was given

** Mann–Whitney *U* test.

## Footnotes

1 COVID-19 Coronavirus Pandemic (2021). [online]. Website https://www.worldometers.info/coronavirus/ [accessed 15 April 2021].

2 T.C. Sağlık Bakanlığı Halk Sağlığı Genel Müdürlüğü COVID-19 (SARS-CoV-2 Enfeksiyonu) Rehberi Bilim Kurulu Çalışması, 25 Mart 2020 (2020). [online]. Website https://hastane.deu.edu.tr/images/hastanemizden-haberler/corona/COVID-19_Rehberi_25.03.2020.pdf [accessed 1 April 2021]
