# Supplementary material for: A COVID-19 first evaluation clinic at a university hospital in Turkey
Source: Turk J Med Sci. 2021 Sep 7;52(1):1–10. doi: 10.3906/sag-2104-152 (PMC10734817; doi:10.3906/sag-2104-152)
Supplement: Supplementary file 1 [file TURKJMEDSCI-52-1-1-Supplementary-Table-1.pdf]

Hacettepe University Adult Hospital COVID-19 Outpatient Clinic Patient Admission and Follow-up Protocol

1- The patient should wear a surgical mask
2- The evaluation will start with the history of the complaints and then physical examination will be performed with appropriate personal protective equipment
3- Check vital signs and oxygen saturation (Patients who need oxygen support should be referred to the emergency room)
4- Take the patient to the sampling room
5- The healthcare worker should wear appropriate personal protective equipment before entering the sampling room
6- Request blood tests, radiological examination and combined oro- nasopharyngeal swab for SARS-CoV-2PCR

A. Blood tests

All patients: Complete blood count, C-reactive protein (CRP)

Patients with a comorbid disease or> 50 years of age: Complete blood count, C-reactive protein, procalcitonin, renal and liver function tests, lactate dehydrogenase, cardiac enzymes, coagulation parameters, if SpaO2 <90%, arterial blood gas, lactate, D-dimer, ferritin

B. Radiological examination

Chest X-ray and, low-dose chest CT without contrast in the following cases

1-If the chest X-ray is diagnostic in a patient with symptoms of lower respiratory tract infection, CT is not required

2-If the chest X-ray is normal in a patient with fever and / or persistent cough, request low-dose thoracic CT (without contrast media).Thoracic CT with a contrast media will be requested if there is suspicion of pulmonary emboli or pulmonary mass.

3 - A protected postero-anterior chest X-ray can be performed in a pregnant women with a consent form

C. Respiratory Viral Panel (For differential diagnosis)

D. Nasopharyngeal sampling for SARS-CoV-2 real-time PCR

After the first evaluation:

PneumoniaX

Blood tests are in normal range ✓

Comorbid diseaseX

PO_2>93 at ambient air, respiratory rate <20/min✓

- Home isolation while waiting for SARS-CoV-2 PCR test results
- Prescribe oseltamivir if thereis a suspicion of influenza
- Recommend to re-admit to the hospital if clinical deterioration occurs

SARS-CoV-2PCR/Chest CT Negative

- Readmission to the hospital is recommended if clinical deterioration occurs
- Home isolation for 14 days if there is a close contact history
- If the patient is a healthcare worker follow the algorithm according to Ministry of Heallth regulations

SARS-CoV-2PCR/Chest CTPositive

Follow 'Hacettepe University Adult and Oncology Hospitals Management of the COVID-19 -Positive patients ' algorithm
