# Supplementary figures and images for: A COVID-19 first evaluation clinic at a university hospital in Turkey
Source: Turk J Med Sci. 2021 Sep 7;52(1):1–10. doi: 10.3906/sag-2104-152 (PMC10734817; doi:10.3906/sag-2104-152)

## Hacettepe University Adult Hospital Management Algorithm for COVID-19 Patients

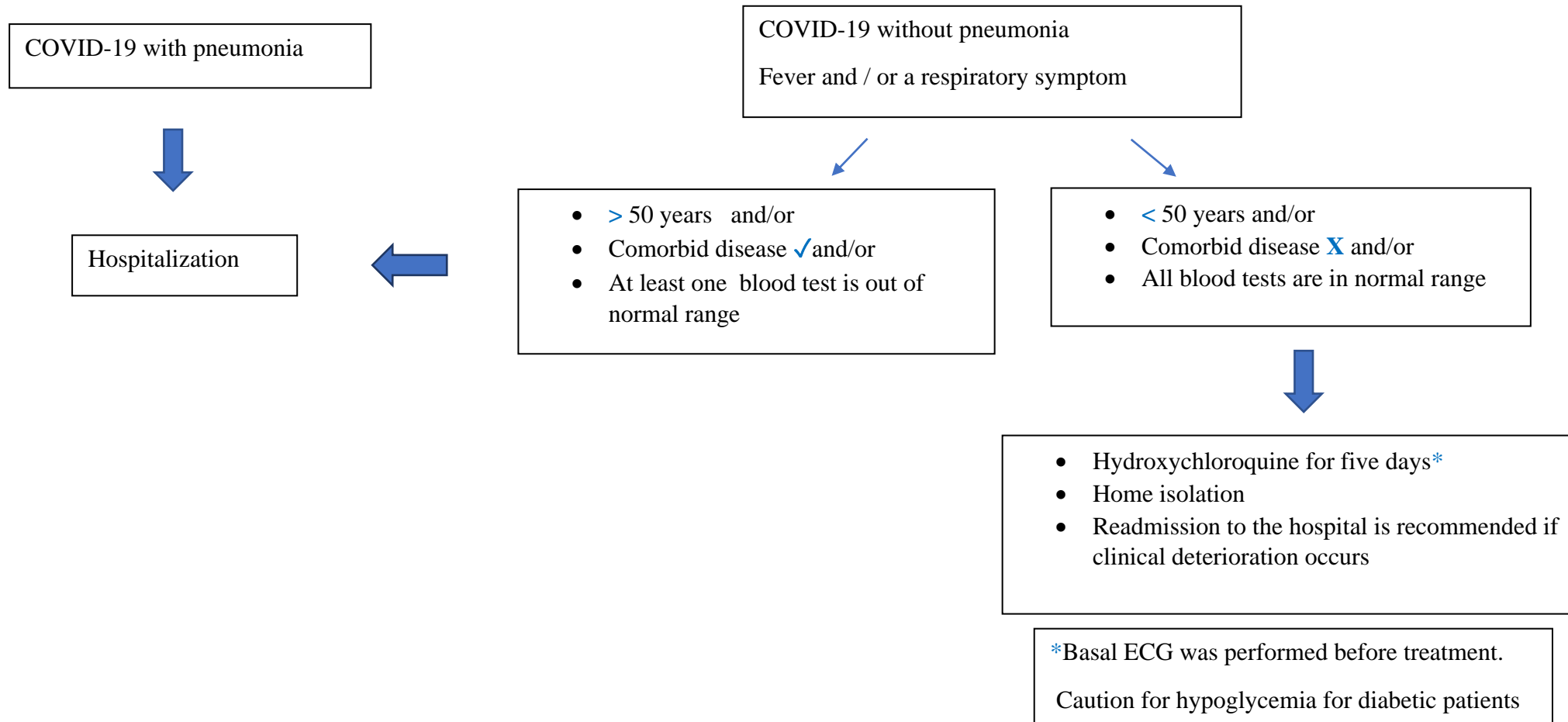

Supplement: Supplementary file 2 [file TURKJMEDSCI-52-1-1-Supplementary-Table-2.pdf]
